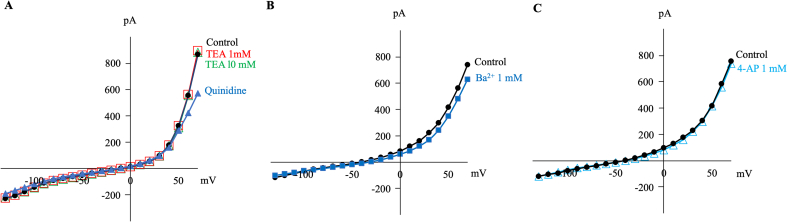# Two-pore domain potassium channel TREK-1 contributes to arachidonic acid-induced Ca^2+^ signaling in human fibroblast-like synovial cells

**DOI:** 10.1016/j.bbrep.2025.102098

**Published:** 2025-06-23

**Authors:** Battulga Khaltar, Futoshi Toyoda, Kosuke Kumagai, Takafumi Yayama, Batchimeg Tsedenbal, Kohei Umeda, Hideki Saito, Naranbat Lkhagvasuren, Mitsuhiko Kubo, Shinji Imai

**Affiliations:** a Department of Orthopaedic Surgery, Shiga University of Medical Science, Otsu, Shiga, 520-2192, Japan; b Department of the Traumatology and Orthopedics, School of Medicine, Mongolian National University of Medical Sciences, Ulaanbaatar, 14210, Mongolia; c Center Research Laboratory, Shiga University of Medical Science, Otsu, Shiga, 520-2192, Japan; d Department of Sports and Musculoskeletal Medicine, Shiga University of Medical Science, Otsu, Shiga, 520-2192, Japan

**Keywords:** Fibroblast-like synovial cells (hFLSs), Arachidonic acid, K2P channels, TREK-1, Spadin, Calcium signaling

## Abstract

Human fibroblast-like synovial cells (hFLSs) are essential in maintaining the structural integrity of the articular cartilage and promoting joint inflammation. These cells are highly responsive to various physical and chemical stimuli, many of which influence cellular processes through intracellular Ca^2+^ signaling and membrane ion channel activity. In this study, we investigated the role of the TREK-1 two-pore domain potassium (K2P) channel as a molecular sensor of arachidonic acid (AA) in FLSs. Patch-clamp recordings revealed an outwardly rectifying K^+^ conductance resistant to conventional K^+^ channel blockers (4-AP and TEA) but sensitive to inhibition by quinidine, a broad-spectrum K2P blocker. Activation of the TREK-1 channel with 4-(2-Butyl-6,7-dichloro-2-cyclopentyl-indan-1-on-5-yl) oxobutyric acid (DCPIB) and ML402 increased this current, and immunocytochemical staining demonstrated TREK-1 expression in hFLSs. AA exposure potentiated the K^+^ current in a concentration-dependent manner and caused hyperpolarization of the resting membrane potential, effects fully antagonized by pretreatment of the cells with spadin, a TREK-1 selective blocker. Fluorescent Ca^2+^ measurements showed that AA-induced variable increase in the intracellular Ca^2+^ concentration ([Ca^2+^]_i_) in different FLSs, and spadin attenuated these responses, reducing the number of cells exhibiting oscillatory and sustained [Ca^2+^]_i_ elevations. In a nominally Ca^2+^-free medium, spadin had no effect, suggesting that TREK-1 channels regulate plasma membrane Ca^2+^ influx. Our findings provide the first electrophysiological and pharmacological evidence for the involvement of TREK-1 channels in AA-induced Ca^2+^ signaling in hFLSs.

## Highlights

• TREK-1 channels as key regulators in human fibroblast-like synovial cells (hFLSs).
• Arachidonic acid activates TREK-1, modulating membrane potential and Ca^2+^ signaling.
• TREK-1 channels contribute to membrane hyperpolarization in response to stress.

## 1 Introduction

Fibroblast-like synovial cells (FLSs) are the predominant synovial cell population in human joints and play a crucial role in maintaining the structural integrity of the articular cartilage. FLSs produce and secrete molecules such as hyaluronic acid, glycoproteins, and cytokines, which are essential for joint lubrication and synovial fluid conservation. However, in chronic inflammatory joint diseases like rheumatoid arthritis (RA), FLSs contribute to cartilage dysfunction. In the RA synovium, FLSs undergo phenotypic changes, leading to hyperproliferative and invasive behaviors [[1], [2], [3]]. Extensive research on RA FLSs has revealed alterations in cell cycle, metabolic homeostasis, signal transduction, and membrane electrical activity [4,5]. As a result, FLSs have emerged as potential drug targets in RA therapy.

Various physical and chemical stimuli, such as mechanical loading, acidification, and proinflammatory mediators, modulate cellular processes in FLSs, often via Ca^2+^-mediated intracellular signaling. This signaling relies on the fine-tuning activity of ion channels [6,7]. Previous patch-clamp studies have identified several Ca^2+^ influx pathways in the plasma membranes of FLSs, including L-type Ca^2+^ [8,9], transient receptor potential (TRP) [10], and acid-sensitive ASIC III channels [6,11,12]. Additionally, K^+^ channel activation is required to produce substantial Ca^2+^ influx, as outward K^+^ conductance hyperpolarizes the membrane potential, enhancing the driving force for Ca^2+^ entry through Ca^2+^ channels [13,14]. The involvement of the Ca^2+^-activated K^+^ currents (mediated by KCa1.1 and KCa3.1 channels) has been suggested [7,15]. Furthermore, recent quantitative polymerase chain reaction (qPCR) analyses have indicated high expression of two-pore domain K^+^ (K2P) channels in FLSs [16], although their functional nature remains largely unexplored.

K2P channels are widely present in various human tissues and organs [[17], [18], [19], [20], [21]], with increasing experimental evidence highlighting their diverse roles in human physiology, as well as in various diseases, including neuronal, cardiovascular, endocrine, and metabolic disorders [[22], [23], [24]]. The human K2P family consists of 15 members, categorized into six subfamilies (TREK, TASK, TALK, TWIK, THIK, and TRESK) based on structure and function. Among these, TREK and TWIK transcripts are most abundant in FLSs [16]. TREK channels (TREK-1, TREK-2, and TRAAK) function as molecular sensors, responding to mechanical forces, temperature, and membrane-derived polyunsaturated fatty acids (PUFAs) like arachidonic acid. These channels mediate outwardly rectifying background leak K^+^ currents to regulate membrane potential [[25], [26], [27]].

In this study, we performed patch-clamp experiments to identify and characterize the electrophysiological and pharmacological properties of TREK channels in human FLSs. Using specific agonists and antagonists, we investigated the role of TREK-1 channels in regulating membrane potential and intracellular Ca^2+^ handling in FLSs.

## 2 Materials and methods

### 2.1 Sample collection

All experimental procedures were reviewed and approved by the Research Ethics Committee of Shiga University of Medical Science (R2017-093). Normal human synovial tissue was obtained from five patients with meniscal tears who underwent arthroscopic resection at the Department of Orthopedic Surgery, University Hospital, Shiga University of Medical Science, between May 2022 and December 2023.

### 2.2 Isolation and cell culture

Resected soft tissue was stored in a 15 mL centrifuge tube with transport media at room temperature (22-25 °C) before processing. In the cell culture laboratory, tissues were washed using phosphate-buffered saline (PBS, Dulbecco's Formula without calcium and magnesium) and digested with 0.5 % collagenase (Type 2; Worthington Biochemical Corp., Lakewood, NJ, USA). The digested tissue was subsequently incubated in a 15 mL centrifuge tube containing Dulbecco's modified Eagle's medium (DMEM; Gibco BRL, Grand Island, NY, USA) enriched with 10 % fetal bovine serum and penicillin-streptomycin-amphotericin B in a humidified atmosphere of 95 % air and 5 % CO_2_ at 37 °C for 4–12 h. After incubation, cell pellets were strained through a 40 μm strainer (BD Falcon, blue) harvested by centrifugation at 432×*g* for 5 min and seeded into a plastic culture Petri dish containing 25 mL DMEM. The medium was changed weekly. The cells were plated and maintained until 80 % confluence; at this point, they formed a homogenous fibroblast population through 3–8 passages [28]. When confluent, the cells were detached from the flask surface using 1 % trypsin-ethylenediaminetetraacetic acid (EDTA), then centrifuged at 432×*g* for 5 min. The resulting pellet was resuspended in fresh medium for subculture and experiments.

### 2.3 Immunocytochemistry - immunofluorescence (ICC-IF)

ICC-IF was used to investigate the localization of Cadherin-11 (CDH11) and TREK-1 channels in FLS. All procedures were performed at room temperature unless otherwise specified. The cells were fixed with 4 % paraformaldehyde for 10 min, permeabilized with 0.1 % Triton X-100 for 10 min, and blocked with 2 % bovine serum albumin (BSA) for 1 h. Samples were incubated overnight at 4 °C with CDH11 antibody (PA5-88399, Thermo Fisher Scientific, Inc, Waltham, MA, USA) to validate synovial fibroblasts and with Anti-KCNK2 (TREK-1) antibody (Alomone Labs; #APC-047, 1:100 in EnVision Flex Antibody Diluent Solution, Dako) to detect TREK-1 expression (1 μg/mL in 0.05 % PBST). The cells were labeled with Goat anti-Rabbit IgG Alexa Fluor® 488 secondary antibody (1:100, 120 min). The nuclei were counterstained with Hoechst 33342 (Thermo Fisher Scientific Inc, Waltham, MA, USA).

### 2.4 Whole-cell patch clamp electrophysiology

FLS cell suspensions were transferred to a recording chamber (0.5 mL volume) on the stage of a Nikon TMD-300 inverted microscope (Nikon, Tokyo, Japan) and allowed to adhere lightly to the glass bottom for 5 min. The chamber was continuously perfused with normal Tyrode solution containing (in mM): NaCl 140, KCl 5.4, CaCl_2_ 1.8, MgCl_2_ 0.5, NaH_2_PO_4_ 0.33, glucose 5.5, and HEPES 5 (pH = 7.4 adjusted with NaOH) at 36 ± 1 °C at a constant rate of 2 mL/min. Whole-cell membrane currents and potentials were recorded using an EPC-8 patch-clamp amplifier and PatchMaster acquisition software (HEKA; Lambrecht, Germany). Pipettes were pulled from glass capillaries using a Sutter P-97 microelectrode puller (Novato, CA, USA), fire-polished by a microforge to achieve a resistance of 2.5–4.5 MΩ when filled with the pipette solution (in mM): aspartate 70, KCl 40, KH_2_PO_4_ 10, MgSO_4_ 1, HEPES 5 and EGTA 5 (pH = 7.2 adjusted with KOH). After equilibration, whole-cell membrane currents were measured using either the voltage step square pulses or a voltage ramp from a holding potential of −40 mV. The voltage ramp protocol (dV/d*t* = ±0.25 V/s) consisted of three phases: an initial depolarizing phase to +70 mV from a holding potential of −40 mV, a hyperpolarizing phase to −130 mV, and a return to the holding potential. The current-voltage (I–V) relationship was measured during the hyperpolarizing phase. All voltages were corrected for a liquid junction potential of −10 mV. In each cell, the current density was calculated by dividing the current amplitude by the cell membrane capacitance (69.74 ± 5.23 pF, n = 40), estimated from the capacitive transients.

### 2.5 Measurement of intracellular Ca^2+^ concentration

Intracellular Ca^2+^ ([Ca^2+^]_i_) concentration was measured at room temperature. FLSs were transferred into eight-well chamber slides filled with normal Tyrode solution, loaded with Fluo-8 AM (a fluorescent Ca^2+^ indicator), and incubated in 5 % CO_2_ at 37 °C for 30 min. The cells were then washed twice with fresh solution. Emitted fluorescence was detected at 488 nm using a confocal Dragonfly microscope (Nikon), and time-lapse images were acquired every 10 s for 8 min. Cells were equilibrated in external solutions containing 1.8 mM or no Ca^2+^. Spadin (250 nM) was applied a few minutes before the experiment, and AA was carefully added to the recording chamber during signal acquisition. Fluorescence intensity was measured in 20 μ m × 20 μ m square “regions of interest,” each representing a single cell, using Imaris software. The fluorescence intensity was normalized and expressed as F/F_0_, where F_0_ is the baseline fluorescence intensity before drug administration.

### 2.6 Statistics and analysis

Data are expressed as mean ± standard error (SE), with the number of cells indicated. Statistical comparisons were performed using Student's t-test or the Wilcoxon test, as appropriate, with a significant threshold set at *p* < 0.05.

## 3 Results

### 3.1 Outwardly rectifying background K^+^ currents in human FLSs

**Fig. 1:**
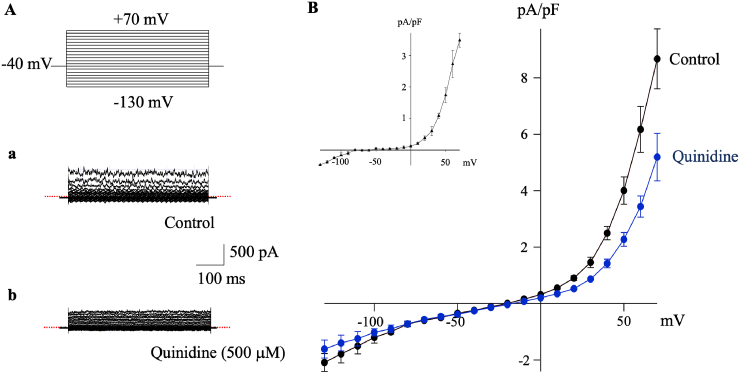
Whole-cell patch-clamp recordings of quinidine-sensitive currents in hFLSs **A.** Representative superimposed whole-cell current traces in response to 500-ms voltage-command steps applied from holding potential of −40 mV to test potentials between +70 mV and −130 mV in control (a) and during exposure to 500 μM quinidine (b). Dotted line (red) indicates zero. **B.** Averaged current density-voltage (I–V) relationships of whole-cell currents recorded in control and during exposure to 500 μM quinidine. Inset: the I–V relationship of the quinidine-sensitive (difference) currents, which exhibits an outward rectification and a reversal potential around −63.3 ± 1.03 mV (n = 6), characterized by a Na^+^/K^+^ permeability ratio of approximately 0.05 when calculated using the Goldman-Hodgkin-Katz equation. hFLSs: Human fibroblast-like synovial cells.

Whole-cell patch-clamp recordings were performed to investigate the membrane current properties in FLSs. Cells were dialyzed with a pipette solution containing 5 mM EGTA (without added Ca^2+^) to minimize contamination of Ca^2+^-activated K^+^ currents. Fig. 1 shows a representative example of current recordings in response to 500-ms voltage-command steps from a holding potential of −40 mV to test potentials ranging from +70 mV to −130 mV. These currents exhibited time-independent background currents with a marked outward rectification (allowing outward current to flow easily) at depolarized potentials (>+20 mV) and a weaker inward rectification (allowing inward current to flow easily) at hyperpolarized potentials (<-80 mV), producing a non-monotonic I–V relationship when the current densities were plotted against the membrane potentials (Fig. 1A and B). The background currents were significantly inhibited by quinidine (500 μM), with 22.8 % and 40.1 % inhibition at −130 mV and +70 mV, respectively (n = 3, p < 0.05). The averaged reversal potential of the quinidine-sensitive current was approximately −63.3 ± 1.03 mV (Fig. 1B, *inset*), indicating preferential blockage of K^+^ carried currents over Na^+^. In contrast, the background current was almost resistant to 4-aminopyridine (4-AP, 5.11 % inhibition at 1 mM) and tetraethylammonium (TEA, 0.29 % and 6.65 % inhibition at 1 and 10 mM, respectively, n = 4), which are known inhibitors of voltage-gated K^+^ (Kv) channels, but was slightly reduced by Ba^2+^ (20.24 % inhibition at 1 mM) (Supplementary Fig. 1). These findings suggest that the background K^+^ currents are consistent with the pharmacological properties of K2P channels, including TREK channels [20,29].

### 3.2 Existence and functions of TREK K2P channels in hFLSs

**Fig. 2:**
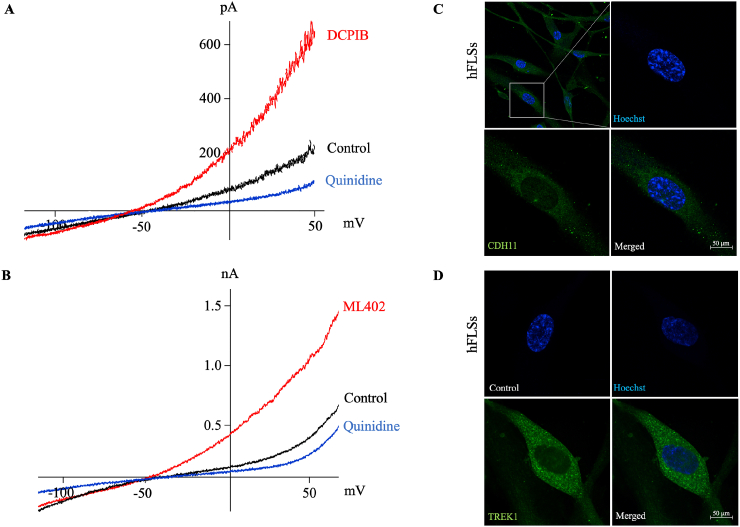
Functional expression of TREK-1 in hFLSs **A.** I–V relationships obtained by a voltage ramp in the absence (black) and presence (red) of DCPIB, and subsequent application of quinidine (blue). **B.** I–V relationships obtained by a voltage ramp in the absence (black) and presence (red) of ML402 and subsequent application of quinidine (blue). **C and D.** Representative immunofluorescence images for validation of synovial fibroblasts with CDH11 (green, C) and positive expression of TREK-1 (green, D). Nuclei (blue, C and D) were stained with Hoechst. Control (D) with a no-primary antibody.

The TREK subfamily of K2P channels consists of three members: TREK-1, TREK-2, and TRAAK. In the experiment shown in Fig. 2, we examined the effects of three potent activators of TREK channels. 4-(2-Butyl-6,7-dichloro-2-cyclopentyl-indan-1-on-5-yl) oxobutyric acid (DCPIB) has been broadly used as a selective blocker of volume-sensitive Cl^−^ currents [30,31] but has recently been shown to activate TREK-1 and TRAAK channels [32,33]. Bath application of DCPIB (20 μ M) markedly increased the outward rectifying K^+^ current (Fig. 2A). ML402 (20 μ M), a thiophene-carboxamide compound reported to selectively activate TREK-1 and TREK-2 (but not TRAAK) [34,35], also potentiated the outward-rectifying K^+^ current (Fig. 2B). In contrast, aprepitant, a TRAAK channel agonist, did not increase the current (Fig. 2C). These results are consistent with a previous qPCR study showing mutually exclusive expression of TREK-1 (*KCNK2*) in hFLSs [16]. Immunocytochemistry analysis with IF staining revealed that virtually all cells were positive for CDH11, a marker of synovial fibroblasts, and TREK-1 (Fig. 2C and D).

### 3.3 TREK channel is activated by arachidonic acid

**Fig. 3:**
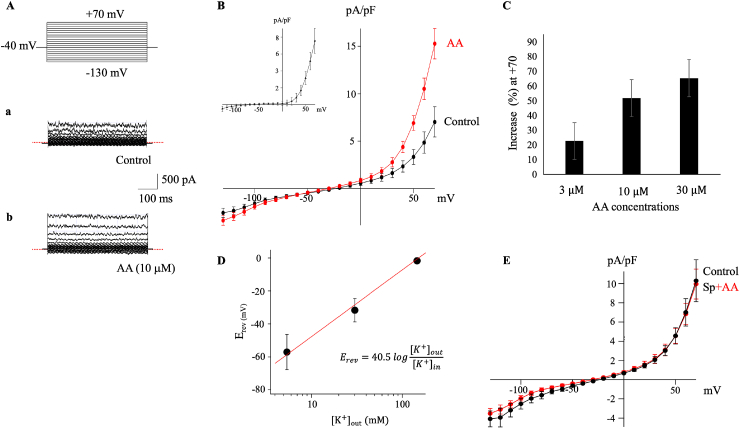
Modulation of TREK-1 channel by arachidonic acid (AA) in hFLSs **A**. Representative superimposed current traces in control (a) and during exposure to 10 μM AA (b). **B.** Averaged I–V relationships in control and during exposure to 10 μM AA. Inset: the I–V relationship of the AA-activated (difference) currents, exhibiting an outward rectification and a reversal potential approximately −56.9 mV. **C.** Concentration-dependent activation measured by percentage increase in the current amplitude at +70 mV before and after application of AA in the same cell. **D.** Effect of extracellular K^+^ concentration ([K^+^]_o_) on the reversal potential (E_rev_) of AA-activated currents. A fitting linear line shows a slope of 40.5 mV per 10-fold concentration change in [K^+^]_o_. **E.** Lack of the effect of AA in cells pretreated with spadin, a selective antagonist of the TREK1 channel.

A hallmark feature of TREK channels is their modulation by PUFAs [34,36]. To assess this property, we examined the response of membrane currents to AA (Fig. 3). AA (3–30 μ M) increased both inward and outward background currents in a concentration-dependent manner (Fig. 3A, B, and D). The I–V relationship of the AA-induced current exhibited strong outward rectification with reversal potentials of −56.9 ± 26.1 mV (Fig. 3B, *inset*, n = 6), similar to that of the quinidine-sensitive currents (Fig. 1B, *inset*). Increasing external K^+^ concentration ([K^+^]_out_) shifted the reversal potential toward positive potentials by 40.5 mV for a tenfold change in [K^+^]_out_, indicating K^+^ as the main current carrier. The AA-induced current was sensitive to block by quinidine but resistant to TEA, 4-AP, and Tram34 (1 μ M), a potent blocker of KCa3.1 (*data not shown*). AA failed to activate the current in cells pretreated with spadin (250 nM), a selective blocker of TREK-1 (Fig. 3E). Spadin did not affect the background conductance or the pre-activated current by AA because of the “down” state-dependent characteristics of TREK-1 inhibition by spadin [37]. Additionally, ruthenium red (50 μ M), a blocker of TREK-2, had no effect on AA-induced current (*data not shown*). These findings indicate that the AA-induced current is largely attributable to the activation of TREK-1 channels in hFLSs.

**Fig. 4:**
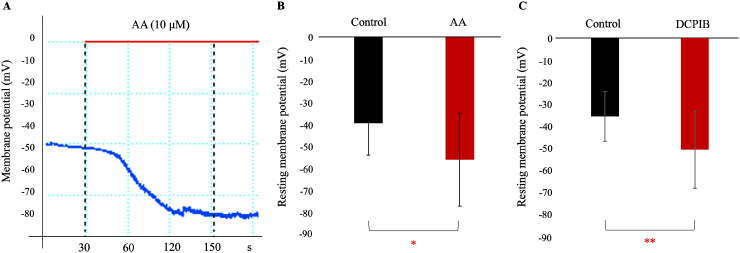
TREK-1 activation hyperpolarizes the resting membrane potential in hFLSs **A**. Typical example of the membrane potential recording in zero-current clamp mode. AA evoked hyperpolarization on the membrane potential. **B and C.** Averaged resting membrane potentials in control and 10 μM AA (B) or DCPIB (C). ∗p < 0.05 (n = 8), ∗∗p < 0.01 (n = 10).

As shown in Fig. 4, we examined the effect of TREK channel activation on the resting membrane potential (*V*_*rest*_) under current-clamp conditions. As reported previously [38], *V*_*rest*_ in hFLSs displayed wide cell-to-cell variation ranging from −10.69 to −51.30 mV (−39.37 ± 14.73 mV, n = 8). This variability was likely due to the intrinsic properties of cells with large input resistances in the voltage range near the reversal potential [6]. Nevertheless, both AA and DCPIB consistently shifted the *V*_*rest*_ toward more negative potentials of −56.15 ± 21.30 mV (n = 8, p < 0.05) and −59.06 ± 19.33 mV (n = 10, p < 0.01), respectively, suggesting that increased K^+^ conductance through TREK-1 activation effectively hyperpolarized the membrane potential.

### 3.4 TREK-1 contributes to AA-induced intracellular Ca signaling in hFLSs

**Fig. 5:**
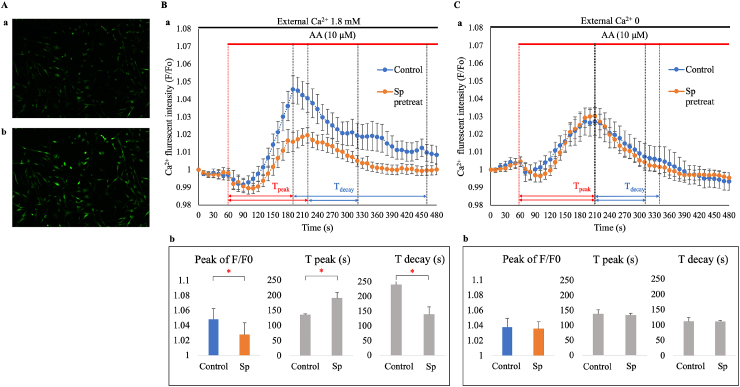
AA-induced intracellular Ca^2+^ mobilization in hFLSs **A.** Fluorescent images of intracellular Ca^2+^ signals in control (a) and after exposure to AA (b). hFLS cells loaded with Ca^2+^ fluorescent indicator Fluo8 **B.** (a) Averaged time courses of changes in relative fluorescent intensity (F/F_0_) of control cells (blue) and cells pretreated with spadin (Sp. orange) in the presence of extracellular Ca^2+^1.8 mM. (b) Averaged peak of F/F_0_, time to peak (T_peak_) and time to decay to 20 % of the peak (T_decay_). ∗p < 0.05, ∗∗p < 0.01. **C.** Experiments performed in extracellular medium with no added Ca^2+^.

AA evokes intracellular Ca^2+^ signaling by promoting both Ca^2+^ release from the internal store and Ca^2+^ entry at the plasma membrane in various cell types [[39], [40], [41]]. We sought to determine if TREK-1 activation by AA is involved in intracellular Ca^2+^ mobilization in hFLSs. To address this, single-cell Ca^2+^ imaging was performed using the Ca^2+^-sensitive dye Fluo-8 (Fig. 5). As shown in Fig. 5A, AA (10 μ M) caused an elevation in [Ca^2+^]_i_ in most cells, although there was cell-to-cell heterogeneity in the timing and pattern of the Ca^2+^ response. The average time course of fluorescent signal changes across approximately 150 cells showed a gradual increase in [Ca^2+^]_i_, followed by a slow decline during exposure to AA (Fig. 5B). This was quantified by the peak of relative fluorescent intensity (F/F_0_), time to peak (T_peak_), and time to decay to 20 % of the peak (T_decay_) in seconds (s). Pretreatment of FLSs with spadin significantly decreased the peak of F/F_0_ (1.048 ± 0.014, n = 6 in control vs. 1.027 ± 0.016, n = 6 in spadin, p < 0.05) and T_decay_ (190.33 ± 19.12 s, n = 6 in control vs. 138.16 ± 25.73 s, n = 6 in spadin, p < 0.01) but increased T_peak_ (134.83 ± 4.40 s, n = 6 in control vs. 238.83 ± 12.86 s, n = 6 in spadin, p < 0.01) (Fig. 5B–a and b).When the Ca^2+^ entry was minimized by lowering external Ca^2+^ from 1.8 mM to nominally Ca^2+^-free (no added Ca^2+^), AA still induced Ca^2+^ responses but with shortened T_decay_ (Fig. 5C). Spadin treatment did not significantly affect these parameters in the absence of extracellular Ca^2+^.

**Fig. 6:**
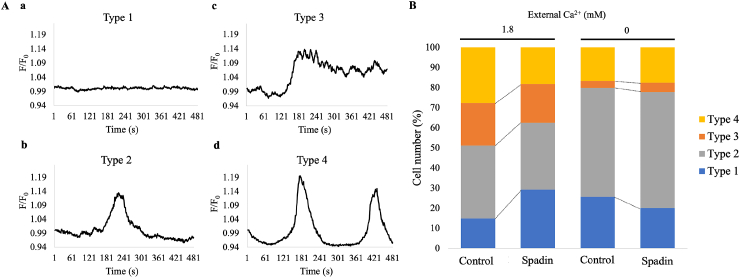
Cellular heterogeneity of Ca^2+^ responses to AA in hFLS **A.** Typical examples for four types of Ca^2+^ responses to AA: no response (a, type 1), a transient increase (b, type 2), sustained increase (c, type 3), and oscillatory increase (d, type 4). **B.** Percentage of the fraction of each cell type shown for control and in the presence of spadin, with or without 1.8 mM Ca^2+^ in the external medium.

To further characterize AA-induced Ca^2+^ mobilization and its modulation by spadin, cell-to-cell heterogeneity was analyzed by classifying individual cells into four types based on Ca^2+^ response patterns to AA (Fig. 6A). A substantial fraction of the analyzed FLSs (166/1114 cells, 14.9 %, Type 1) showed no response. Among the responding cells, three distinct patterns of [Ca^2+^]_i_ signals were observed: a transient increase (405/1114 cells, 36.35 %, Type 2), a sustained increase (235/1114 cells, 21.09 %, Type 3), and an oscillatory increase (308/1114 cells, 27.64 %, Type 4). Spadin decreased the fraction of Type 4 cells (233/1286 cells, 18.11 %) and increased the fraction of Type 1 cells (376/1286 cells, 29.23 %). Removal of external Ca^2+^ strongly reduced the fractions of Types 3 and 4 and increased those of Types 1 and 2, suggesting that sustained and oscillatory Ca^2+^ responses to AA are primarily dependent on Ca^2+^ influx via the plasma membrane. Spadin did not significantly alter the fractions of any cell types under nominal Ca^2+^-free conditions (at 0 Ca, Fig. 6B). These results suggest that TREK-1 plays a role in regulating intracellular Ca^2+^ dynamics in response to AA by modulating Ca^2+^ entry through the plasma membrane.

## 4 Discussion

This study provides the first electrophysiological and pharmacological evidence for the presence of TREK-1 channels in hFLSs. Using patch-clamp experiments, we identified a TEA-insensitive and quinidine-sensitive outward-rectifying background K^+^ current (Fig. 1), which was potentiated by TREK-1 channel activators (DCPIB and ML402, Fig. 2). Additionally, this current was activated by AA and was sensitive to the TREK-1 channel-selective inhibitor, spadin (Fig. 3). These properties align with those previously reported for TREK-1 channels in various cell types [34,37,42]. Furthermore, our data demonstrate that TREK-1 channels are involved in AA-induced membrane hyperpolarization and intracellular Ca^2+^ signaling in hFLSs (Fig. 4, Fig. 5, Fig. 6). TREK channels respond to various stimuli, including AA, mechanical force, intracellular pH changes, and temperature variations [26,27,43]. The identification of TREK-1 as a sensor molecule in hFLSs will facilitate the understanding of how stress signals are sensed and transduced into cellular processes and functions, with implications for human joint physiology and diseases.

The K2P channel family comprises 15 distinct molecular members that generate similar background leak K^+^ currents but exhibit different responses to specific stimuli [21,23]. The molecular identification of K2P channels is crucial for understanding their distinct roles in various tissues and cell types. In this study, we focused on the TREK subfamily of K2P channels, owing to the availability of selective agonists and antagonists. The findings highlight the predominant activity of TREK-1, providing a starting point in elucidating the molecular background of K2P channels in hFLSs. However, the possibility that other K2P channels are expressed and function in these cells cannot be excluded. Kondo et al. [16] reported the expression of TREK-1 (*KCNK2*) and, to a lesser extent, TWIK-2 (*KCNK6*) in hFLSs. While the TWIK channel family displays low functional activity when expressed alone, it plays a significant role when forming heterodimers with TREK-1 [44]. Similarly, TREK-2, TRESK, and TRAAK have been shown to form heterodimeric complexes with TREK-1 [45]. Therefore, heterodimerization could increase the functional diversity of K2P channels in hFLSs, further enhancing their role in cellular processes.

TREK K2P channel-mediated currents typically exhibit outward rectification, a property that helps prevent excessive depolarization of the membrane potential. This outward rectification maintains high input resistance over a wide range of membrane potentials, from *E*_K_ to approximately −10 mV, thereby contributing to passive membrane properties in FLSs [6]. The recordings of resting membrane potentials exhibited large cell-to-cell variability. Kolomytkin et al. [38] reported a bimodal distribution of resting membrane potentials in FLSs, suggesting a switch between two electrophysiological states in response to pathological stress signals and cytokines. The current study showed that TREK activation caused striking membrane hyperpolarization, raising the possibility that TREK channel activity associated with various stress responses could contribute to the biostability of FLS membrane potentials in response to stress.

Among the various regulatory stimuli affecting TREK channels, we focused on activation by AA, given its involvement in the pathogenesis of cartilage diseases such as RA and OA [46,47]. AA is well-known for its role in inflammatory responses and cellular apoptosis, primarily through its oxidized metabolites produced by cyclooxygenase and lipoxygenase pathways [[48], [49], [50]]. Additionally, AA can directly modulate ion channels and receptors, initiating cellular Ca^2+^ signaling [40,51]. In line with these findings, the present study showed that AA evoked intracellular Ca^2+^ elevation in hFLSs, likely due to combined activation of Ca^2+^ release from the endoplasmic reticulum and Ca^2+^ influx pathways such as TRPV4 channels and Orai3-mediated store-operated Ca^2+^ entry (SOCE) [10,51,52]. We observed that spadin effectively altered AA-mediated Ca^2+^ dynamics only in the presence of extracellular Ca^2+^, suggesting an interplay between TREK channels and Ca^2+^ influx in regulating Ca^2+^ signaling. Previous studies have suggested that KCa3.1 channels are tightly coupled with Ca^2+^ channels in a positive feedback loop, where KCa3.1 activation hyperpolarizes the membrane, thereby increasing the driving force for Ca^2+^ influx [7,53]. A fundamental difference exists in the gating mechanism between TREK and KCa3.1; TREK channels are directly activated by AA, whereas the opening of KCa3.1 is triggered by intracellular Ca^2+^. However, both K^+^ channels can be activated by AA and mediate Ca^2+^ signaling, suggesting that they complement the regulation of FLS membrane dynamics and inflammatory responses.

Accumulating evidence suggests that Ca^2+^ signaling plays a crucial role in FLS activation, especially in the inflammatory environment of RA. Excessive Ca^2+^ influx triggers calcineurin/NFAT signaling, thereby leading to FLS hyperproliferation and aberrant secretion of inflammatory mediators, such as cytokines (interleukin-1β, interleukin-6), granulocyte-macrophage colony-stimulating factor (G-CSF) [[54], [55], [56]], and receptor activator of nuclear factor κB ligand (RANKL) [57]. Thus, targeting Ca^2+^ influx pathways may contribute to treating pathological conditions in RA-FLs. Experimental approaches have suggested that inhibiting Ca^2+^ influx through SOCE, TRP channels, and P2X7 purinergic receptors could suppress inflammatory responses [12,58,59]. Additionally, inhibiting KCa channels also presents an effective way to reduce the invasive phenotype of RA-FLS and attenuate disease severity in animal models of RA [60], highlighting an alternative strategy for moderately reducing Ca^2+^ influx without disrupting Ca^2+^ homeostasis. Although the association of TREK1 with arthritic diseases pathogenesis remains unclear, recent research suggested its involvement in idiopathic inflammatory myopathies [61]. Thus, whether TREK1 could be a potential target for treating RA remains a warranted question.

Although this study demonstrates that spadin effectively suppresses TREK1 function, the non-selective effects of this agent cannot be fully ruled out. Experimental approaches, such as gene silencing in animal models, may present useful approaches to clarify the involvement of TREK1 in RA pathogenesis. Given the interplay between ion channels and AA-mediated signaling, future studies should explore their contributions to synovial fibroblast function and pathophysiology, particularly their potential as therapeutic targets in inflammatory joint diseases.

## CRediT authorship contribution statement

**Battulga Khaltar:** Writing – original draft, Methodology, Investigation, Data curation. **Futoshi Toyoda:** Writing – review & editing, Validation, Methodology, Investigation, Funding acquisition, Data curation, Conceptualization. **Kosuke Kumagai:** Writing – review & editing, Validation, Methodology, Investigation, Funding acquisition, Data curation, Conceptualization. **Takafumi Yayama:** Supervision. **Batchimeg Tsedenbal:** Resources, Methodology, Investigation, Data curation. **Kohei Umeda:** Investigation. **Hideki Saito:** Investigation, Data curation. **Naranbat Lkhagvasuren:** Supervision, Methodology, Data curation. **Mitsuhiko Kubo:** Supervision. **Shinji Imai:** Writing – review & editing, Supervision.

## Funding

This work was partly supported by the 10.13039/501100001691JSPS [grant numbers KAKENHI: 21K06781 and 24K10025 to FT, JSPS KAKENHI: 20K09407 to KK].

## Declaration of competing interest

The authors declare that they have no known competing financial interests or personal relationships that could have appeared to influence the work reported in this paper.

## Figures and Tables

**figs1:**